# "They see me as mentally ill": the stigmatization experiences of Chinese menopausal women in the family

**DOI:** 10.1186/s12905-023-02350-y

**Published:** 2023-04-19

**Authors:** Qiong Li, Jintu Gu, Jianyuan Huang, Pei Zhao, Chenliang Luo

**Affiliations:** 1 School of Public Administration, Hohai University, Nanjing, 211100 China; 2 Department of Sociology, Hohai University, Nanjing, China; 3 Research Center for Environment and Society, Hohai University, Nanjing, China; 4 Yangtze Institute for Conservation and High-quality Development, Jiangsu Research Base, Nanjing, China; 5 Jiangsu Province Hospital, Nanjing, 211100 China; 6 Shanghai Pudong New Area Tax Bureau, State Taxation Administration, Shanghai, 200120 China

**Keywords:** Menopause, Women, Family, Stigmatization experience

## Abstract

**Background:**

Menopausal women are regarded as “abnormal people” in China and are often discriminated against and ostracized, especially in the privacy of their homes. However, research on the stigmatization of menopausal women in China is limited. The aim of this study is to explore and describe the stigmatization experiences of Chinese menopausal women in the family and their feelings about these experiences.

**Methods:**

A phenomenological qualitative research design involving in-depth semi-structured interviews was selected. Our data analysis adopted Colaizzi's methodology.

**Results:**

Fourteen menopausal women participated in this study. Four themes and 12 subthemes emerged: (1) violent treatment (verbal and physical violence); (2) lack of attention and companionship (lack of understanding of physical and psychological suffering, neglect of the value of labour and difficulty finding someone to talk to and accompany them); (3) coping struggles (keeping quiet, fighting back, changing inappropriate perceptions and developing a menopausal transition management plan); and (4) despair (deep-rooted perceptions, restrictions on travel and consumption, and unknown "healing" times).

**Conclusion:**

Our results suggest that Chinese menopausal women suffer physically and mentally within their families. The stigma of menopause is both a symptom of the broad societal lack of knowledge regarding menopause and a reflection of the patriarchal oppression of women in a specific cultural context. Accordingly, this study can help menopausal women and society in general better understand the former’s stigmatization experiences and amplify their inner voices. Moreover, it can serve as a reference for the formulation of menopause-related health policies in China and for advocating and promoting humanistic care for menopausal women.

## Introduction

Menopause is the period of life when the menstrual cycle ceases and is caused by a decrease in the production of the ovarian hormones oestrogen and progesterone [1]. Menopause is an inevitable stage in human physical development filled with novel experiences, tasks and challenges. Women in the menopausal transition are often subject to a variety of symptoms that can negatively affect their daily life, physical and mental health and quality of life [2, 3]. According to one report, the number of women over 50 in China was approximately 130 million in 2000 and is expected to exceed 280 million by 2030, implying that Chinese women may be at risk of postmenopausal disease for at least one-third of their lives [4]. Accordingly, Chinese menopausal women represent a population that merits more in-depth research.

The extensive international literature on menopause currently focuses on the treatment and management of physical symptoms. Most women experience various physical and psychological symptoms during the menopausal transition, e.g., fatigue, sexual problems, muscle and joint pain, insomnia, mood swings, depression, reduced confidence and self-esteem, forgetfulness and difficulty concentrating [5–8]. According to previous studies, hot flashes and insomnia are the most common menopausal symptoms among women in Western countries [9], while fatigue, insomnia, irritability, palpitation and depression are the five most common symptoms in Chinese women [10]. Previous studies have shown that the incidence and severity of menopausal symptoms are related to various physiological factors (i.e., reproductive hormones and genetics), sociocultural characteristics (i.e., country, race and age), health status (i.e., chronic diseases, overweight or obesity) and lifestyle (i.e., diet, exercise, smoking and drinking) [11, 12]. The involvement of these factors suggests that the incidence and severity of menopausal symptoms can be controlled to some extent. With the increasing awareness of healthy ageing, women are paying increasing attention to the treatment and management of menopausal symptoms. Menopausal hormone therapy [13], acupuncture [14] and Danggwijagyaksan (DJS) [15] have been shown to be clinically effective in relieving menopausal syndrome. At present, traditional Chinese medicine is widely used in Asia to treat menopausal syndrome and can not only significantly relieve symptoms and improve women's quality of life but is also more economical and safer [16]. In addition, nonpharmacological approaches such as healthy eating, physical exercise, stress management, and catharsis have been found to be helpful in managing menopause [17–19]. For example, one study revealed that overweight or obese individuals were at a higher risk for menopausal symptoms than those at a normal weight, and this risk increased with high dietary fat intake [20]. Therefore, controlling and managing diet is an important way to alleviate menopausal symptoms. Additionally, vibration therapy, chromotherapy, melotherapy, aromatherapy and aeroionotherapy can alleviate menopausal disorder symptoms among patients with metabolic syndrome [21]. In short, these studies have contributed greatly to our understanding and management of menopause.

However, there are relatively few studies on the life experiences of menopausal women. Studies conducted in the Western Province of Sri Lanka [22], Iran [23, 24], and Southeastern Louisiana [25] shed light on women’s understanding and experience of menopause and its impact on their lives. Some studies focused on the menopausal experience of women with diseases. For example, some studies have investigated how autistic women deal with major life changes during the menopausal transition in middle age [26] and their understanding of the positive and negative aspects of menopausal changes [27]; other studies focus on how bipolar affective disorder patients experience mood changes during menopause and the impact on their treatment decisions [28]. In addition, some scholars have called for research on migrant and refugee women related to sexual embodiment, including menopause, to promote sexual and reproductive health and understand gender subjectivity. Thus, several studies have described how migrant and refugee women who settle in Australia or Canada access, understand, evaluate, and use health resources related to menstrual suspension [29, 30]. The investigation of the menopausal experience of immigrant and refugee women not only helps to understand the similarities and differences in the menopausal structure under various cultural backgrounds but also helps these women to obtain and benefit from information related to menopausal health-promoting behaviour. There is also some literature focusing on the work practices of menopausal women. Conducting interviews and observing the daily work of female employees in the United Kingdom and other countries revealed the stigmatization of menopause at work and how women experience and manage menopause at work, allowing a deeper understanding of women’s experiences. Nevertheless, while these studies provide important information, a comprehensive understanding of the lived experiences of menopausal women in China remains lacking. At present, we know little about the real situation of menopausal women in China and how they cope with this transition period.

Even in the Chinese database, the focus of attention on menopausal women is more on their mental health status [31, 32]; the reproductive health status of rural menopausal women is also a focus [33]. Scholars have put forward some suggestions for promoting the development of reproductive health services for rural menopausal women according to their needs [34]. In addition, some scholars are committed to comparing the menopausal symptoms of women from different ethnic groups. For example, menopausal symptoms and medical-seeking behaviours of Mosuo and Han women have been studied [35], as has anxiety and depression in Korean and Han menopausal residents [36]. Notably, menopause is considered a negative concept with social, gender and cultural connotations concerning a specific stage in a woman's life cycle and is used in negative references to a group of women, e.g., moody, nagging, sensitive, suspicious, incomprehensible or unwoman like [37], which bear strong stigmatizing connotations. Given that stigma can undermine the self-esteem and self-efficacy of stigmatized individuals [38], rendering them victims of social exclusion, stigma research should focus on how stigmatized individuals are devalued, prejudged and negatively stereotyped to better understand this phenomenon [39]. Numerous studies have documented the stigmatization of people living with HIV [40] and mental illness and their negative impacts on their lives [41]. However, the experiences of menopausal women who are deemed “abnormal” and sometimes even mentally ill by the Chinese public have not yet been investigated in detail. Some studies suggest that the cultural norms, social influences and personal perceptions associated with menopause may influence how each woman experiences her menopausal transition [42]. Therefore, the real-life experiences of menopausal women in China merit in-depth research.

In summary, although a relatively large number of studies have been conducted on the physical symptoms, mental health and treatment options of menopausal women, knowledge regarding the real-life experiences of Chinese menopausal women is limited. It is important to listen to menopausal women's voices, especially concerning the family interactions that occur in the intimate setting of their homes and their internal experiences, and to amplify them. Accordingly, the aim of this study is to explore and describe the stigmatization experiences of menopausal Chinese women in the family setting and their feelings about these experiences.

## Method

### Design

A phenomenological qualitative research design involving in-depth semi-structured interviews was selected. This method is particularly effective in exploring complex phenomena, their nature and their meaning for those who experience them. This approach thus enabled the researcher to conduct a detailed study of menopausal women's experiences of stigma in the family. Our data analysis is based on Colaizzi's method, which provides detailed and sequential steps for improving the reliability and dependability of results.

### Participants

We used purposive sampling to obtain a heterogeneous sample for exploring menopausal women's experiences of stigmatization in the household. The sample size was determined as follows: after data saturation was reached, two additional menopausal women were interviewed. If there were no new topics emerging, further recruitment was terminated. Participants were recruited from six communities in Nanjing, an eastern city in China. Our inclusion criteria were as follows: (1) female; (2) no menstruation in the past 12 months; (3) living with family members, e.g., a spouse or children; and (4) willing to share their life experiences, especially their family interactions. Our exclusion criteria were as follows: diagnosed with terminal illness or severe mental illness.

Fourteen menopausal women aged 48 to 53 years participated in this study. Most of them listed housewife as their occupation (*n* = 4), were married (*n* = 11), had completed primary school (*n* = 6) and lived with their husbands and sons (*n* = 3). Their duration of menopause ranged from 14–25 months (Table 1). To maintain confidentiality, participants' real names were replaced with case numbers.

**Table 1:** Demographics and menopausal status of study participants

Participant ID number	Age (years)	Occupation	Marita status	Education level	Living with	Duration of menopause (months)
1	48	Factory staff	Married	Illiterate	Husband, son	14
2	52	Factory staff	Widowed	Primary school	Son, daughter-in-law, granddaughter	21
3	48	Enterprise accountant	Married	High school	Husband, daughter, son-in-law	15
4	51	Housewife	Widowed	Primary school	Son, daughter-in-law, grandson	14
5	52	Self-employed	Married	Illiterate	Husband, daughter, son-in-law, granddaughter	20
6	53	Housewife	Married	Primary school	Father-in-law, husband, son, granddaughter	25
7	51	Teacher	Married	College	Husband, son	15
8	52	Housewife	Married	Illiterate	Husband, daughter, son-in-law, grandson	24
9	50	Housewife	Married	Primary school	Husband, son, daughter-in-law, grandson	15
10	49	Nurse	Married	Junior College	Husband, son	17
11	51	Self-employed	Divorced	Primary school	Daughter	19
12	51	Hotel room cleaning staff	Married	Illiterate	Husband, daughter	17
13	49	Factory staff	Married	Primary school	Husband, daughter	15
14	50	Insurance salesperson	Married	Junior High school	Mother, husband, son	15

### Data collection

Ethical approval for the study was obtained from the Ethics Review Committee of the Jiangsu Province Hospital (2023-SR-012). Data were collected in individual in-depth semi-structured interviews. The interviews were conducted between March and August 2022 at times and locations chosen by the participants (26 interviews were conducted, averaging 63 min in length), including Chinese restaurants, cafes, parks, and participants' homes. At the beginning of each interview, the researcher gave the participant a consent form and asked her to read it carefully. If the participant agreed to its terms, she signed it. This consent form included information about the study, the right to withdraw from participation and participant confidentiality. All the interviews were recorded with participants’ consent and transcribed by the first and second authors within 24 h of their conclusion. These transcripts were then sent to the participants, who were invited to confirm their accuracy. Notably, we also obtained informed consent from the husbands, sons or daughters of the four illiterate study participants.

We also designed an interview topic guide. Participants were asked the following questions to encourage them to share their experiences: "How do your family members feel about menopause? How does this make you feel? "; and "How would you describe your family members' interactions with you once you were in the menopausal transition? What changes took place? How did these changes affect you?" A professor (J.G.) reviewed the interview guide, corrected any inappropriate questions and wording, and added the following prompt: "Please talk specifically about how you have coped with these changes."

### Data analysis

Following Colaizzi (1978), our narrative analysis of each interview entailed seven steps: (1) read the transcript to familiarize yourself with the interview and understand it; (2) find and extract sentences from the transcript that relate to menopausal stigma; (3) formulate meaning(s); (4) divide all meanings into categories, theme clusters and themes; (5) define all emergent themes in an exhaustive description; (6) describe the basic structure of the focal phenomena; and (7) return the results of the study to the participants to determine their accuracy [43].

Each transcript was analysed by the first and second authors to produce a preliminary analysis. Afterwards, all the coresearchers discussed the appropriateness of the former two authors' analysis while reviewing the transcript. If a researcher had a different opinion or suggestion about the results of the analysis, the discussion continued until total consensus was reached. Finally, we also invited five menopausal women with similar characteristics to our participants to examine our findings; they agreed that our results accurately reflected their experiences.

## Results

The following four themes emerged from the categorized interview data: (1) violent treatment; (2) lack of attention and companionship; (3) coping struggles; and (4) despair. Each theme comprises several subthemes that collectively describe the stigmatization experiences of menopausal women in the family (Table 2) and is described in detail below.

**Table 2:** Themes and subthemes of the stigmatization experiences of menopausal women in the family in China

Theme	Subtheme
Violent treatment	Verbal violence
	Physical violence
Lack of attention and companionship	Lack of understanding of physical and psychological suffering
	Neglect of the value of labour
	Difficulty finding someone to talk to and accompany them
Coping struggles	Keeping quiet
	Fighting back
	Changing inappropriate perceptions
	Developing a menopausal transition management plan
Despair	Deep-rooted perceptions
	Restrictions on travel and consumption
	Unknown "healing" times

### Theme 1: violent treatment

The first theme consists of two subthemes that collectively represent the different levels of violent treatment that participants experience in their homes.

#### Subtheme 1: verbal violence

All participants described their experiences of verbal abuse or discrimination by family members due to menopause. As one participant recalled, *"Once, I felt much discomfort in my cervical spine while doing housework, so I complained about it. My husband heard me and scolded me, 'You are so mentally ill! If you're so sick, go jump off a building and die, and you will be relieved! And then, we will have peace in this house!'… Since I went through menopause, he's been so disgusted with me; he used to call me crazy, and he used to curse me to death. No matter how, normal people will be forced into mental illness by him." (Case 5)*

Similar comments were as follows: *"The doctor said I was uncomfortable all over because of menopause and told me it was a natural biological phenomenon. However, my husband thinks it is a disgrace and calls me a spoilsport" (Case 3); "They treat me like I'm mentally ill, like I'm a sinner in the family" (Case 6);* and, *"All I have to do is nag slightly more in the house and my son calls me mentally ill… He did not say anything like that before I went through menopause" (Case 1).*

Clearly, the participants' family members had negative attitudes towards menopause, deeming it almost equivalent to "craziness" or "mental illness," and often employed similar terms to verbally abuse the participants.

#### Subtheme 2: physical violence

Some of the more serious acts of violence experienced by the participants are documented in this subtheme. One of the participants, whose menopause coincided with the initiation of her husband's retirement, expected to receive understanding and love from him regarding her physical and psychological discomfort caused by menopause; instead, she was subjected to physical violence by him:

*"After I went through menopause, my husband became significantly worse towards me and often interrupted me when I was talking. When I was talking to friends and neighbours, he would say, 'Do not listen to her; she's menopausal and out of her mind'. Once, I got very angry and got into an argument with him; he rushed into the kitchen, picked up a pan, banged it on my head and then slapped me." (Case 14)*

Several other participants also experienced similarly violent treatment, as in the following narrative:

*"Once, I experienced much discomfort in my joints and wanted my son to take me to the hospital to have them checked again. My husband forbade it, telling me not to interfere with my son's work. I was in so much pain that I did not listen to him and picked up my mobile phone to call my son. He rushed up, grabbed my phone, smashed it on the ground and then kicked my left ankle, cursing, 'Do not you dare call him! I will kick you to death!’ I want to cry just thinking about it... However, he also wanted what's best for his son, and he did not want me to disturb his son at work; he had good intentions." (Case 9)*

### Theme 2: lack of attention and companionship

Most participants reported that they lacked attention from and companionship with family members. This theme comprises three subthemes that collectively reveal the details regarding the participants' lack of attention and companionship in the family.

#### Subtheme 1: lack of understanding of physical and psychological suffering

The first subtheme indicates that participants suffered both physical and psychological pain from not being understood or cared for by family members: *"Whenever I come back from the hospital after a check-up, my son and daughter-in-law never ask me what the doctor said; no one cares about me" (Case 2); "How can they know how I feel? I am dying of pain in my body. They see me standing in front of them nicely; how can they believe that I am uncomfortable in my body? Perhaps I must lie there and not be able to move before they will truly understand my pain." (Case 3)*

Other participants highlighted that they experienced severe physical symptoms during their menopausal transition but were questioned by family members who thought they were faking these or delusional because these symptoms were not pathological:

*"I feel so tired sometimes, but my son asks me if I'm faking it." (Case 7)*

*"I felt unwell everywhere, but no amount of examination could determine what was wrong; I just felt uncomfortable. They just said it was a hallucination and asked me if I was too idle to think about it... The pain is not in them, so they do not understand my pain." (Case 1)*

*"The doctor said that menopause is when you have [pain] all over the place, but my husband thinks I have paranoia and told me to go to a psychiatric unit, which made me feel very angry and sad." (Case 6)*

#### Subtheme 2: neglect of the value of labour

Whether our participants were housewives or working women, they all agreed that household chores were respected and appreciated. However, some of the participants were not treated in an ideal way. This second subtheme is supported by their voices as follows:

*"In fact, I do not feel well in my joints all over my body; I have panic attacks, and I often feel very tired. However, I still make three meals a day for them, keep the house clean and tidy, and I go to the vegetable market before 6:30 am to buy food. In addition, I have to take care of my three-year-old grandson all day long. They sit on the sofa and play on their phones while I work, and they never praise me for anything I do well. Whenever I do something badly, they say 'You're menopausal!' This means that I have a bad brain." (Case 4)*

One participant stressed that even though she functioned as a nanny in her family, they did not provide her the dignity of a nanny:* "Even if she is a nanny, she still has a salary of three to four thousand per month, and you cannot just insult her. Unlike me, I'm at home every day caring for cattle and horses. Not only does menopause make me physically uncomfortable, but I am scolded by them if I do not do my chores well… I'm now living worse than a nanny." (Case 8)*

Hence, these accounts clearly demonstrate that some of the participants had endured the physical symptoms of menopause while performing household chores without being respected and recognized and that such work was not valued by their family members. Moreover, their status in their family is not equal to that of other family members.

#### Subtheme 3: difficulty finding someone to talk to and accompany them

The third subtheme reflects the difficulty most participants experienced finding someone to talk to and be with at home. Some participants indicated that their family members were not willing to listen to them when discussing their feelings. The following excerpts reflect this reality:

*"My husband does not want to communicate with me at all; he's annoyed with me. I say, ‘I'm not feeling well,’ and he just says, 'What's the point of talking to me? If you're not feeling well, go to the hospital'... Ugh! All my colleagues are more considerate than my husband." (Case 13)*

*"When I mention menopause, my husband says he's tired and wants a quiet space, but if his friends call and invite him out for dinner or a poker game, he's happy to go... In fact, he just does not want to talk to me." (Case 1)*

*"Whenever I confide in my son about my physical symptoms, he acts impatient and tells me to shut up, to stay away from him and not to disturb him, even if he's just on his phone.” (Case 4)*

Other participants also stated that they sometimes did not feel well and wanted a family member to accompany them to the doctor but struggled to find someone who would do so:* "Once, in the middle of the night, I suddenly had a panic attack, and my heart was beating particularly fast. I asked my son to take me to the hospital quickly, but he said, 'Everyone has their own things to do; you are making me tired like this. Cannot you go to the hospital?'" (Case 9); "Who will go to the hospital with me? My son works abroad, and my daughter works in a bank; they are both very busy. My daughter gets off work around midnight every day and even has to work overtime on weekends." (Case 11)*

### Theme 3: coping struggles

The third theme showcases menopausal women’s coping struggles with regards to the stigma of menopause and their experiences. Its four subthemes are keeping quiet, fighting back, changing inappropriate perceptions, and developing a menopausal transition management plan.

#### Subtheme 1: keeping quiet

The first subtheme illustrates the use of silence as a coping strategy by some participants. To avoid more violent conflicts, some participants chose to be silent and tolerant when family members insulted them with words such as "mentally ill" or "crazy":

*"He can scold if he wants to; there is nothing I can do to shut him up… nothing to care about—he scolds me twice and this thing is over. I'm used to it." (Case 8)*

*"They treated me like I was mentally ill, and I was angry at first, crying several times. Now, when they call me names like that, I do not argue with them because even if I did, it would not do me any good; it would just get worse and worse." (Case 6)*

Other participants stated that when they gather to discuss family matters, they often do not receive a response or are ridiculed when they express their views or share their opinions. As a result, they often choose to remain silent and defer to the decisions of their family members:

*"Even if I express my ideas and they do not approve of them, it is fine; however, they still make fun of me for not being in my right mind because I'm menopausal... They also rarely consult me about some family decisions... It is like I'm not treated like a human being because I'm menopausal." (Case 9)*

*"What ideas can I have? My idea is to listen to them, and what I say does not carry any weight anyway." (Case 1)*

In addition, some participants at times desired to speak, yet even if they did, they would not be cared for and perhaps insulted. Over time, it became increasingly rare for them to confide in their families: *"I do not talk to them anymore about what is physically uncomfortable, and when I do, I must be scolded. Therefore, I might as well keep it to myself" (Case 2).*

Therefore, remaining silent does not entail that these participants approve of the humiliation and discrimination they have been subjected to by their family members; rather, they have sought to minimize the number of times they are treated violently. This is a clear reflection of the dilemma menopausal women face in the family.

#### Subtheme 2: fighting back

The second subtheme showcases some participants' experiences defending their dignity with verbal warnings to family members or by leaving their home:

*"When they first discriminated against me, I warned them that if they called me names, I'd go toe-to-toe with them and that I would never put up with them." (Case 7)*

*"Once, they interrupted me, and I fought back. I warned them not to treat me like this if they still thought I was part of the family, or we would go our separate ways." (Case 4)*

One participant also recounted her experience fleeing her home:

*"A friend of mine told me about a gathering of friends where my husband said, in front of them, that I had become mentally unbalanced after menopause and that he wanted to divorce me. I was very angry when I heard that and felt very humiliated. I thought to myself, ‘Since he wants a divorce, I will leave.’ Then, I packed my bags and moved to my sister's house... Later, my mother involved herself in this and urged him to come and apologize to me and take me home. When he came, my sister and I warned him together. I said, 'If there is a next time, I will never go back.'" (Case 14)*

#### Subtheme 3: changing inappropriate perceptions

Some participants also worked to correct the inappropriate perceptions of menopause among their family members; they recognized that many of their ongoing difficulties stemmed from family members' perceptions of menopause.

Several of the participants were highly educated or medical professionals and therefore had a more scientific and comprehensive understanding of menopause than the general population. These participants knew that menopause is a normal physiological phenomenon unrelated to mental illness and that it is a misconception to associate them with one another. Hence, they have educated their family members about menopause and helped them view it as a natural physical phenomenon and thus to care for women in the menopausal transition:

*"I explained to my husband and son my medical knowledge of menopause and told them not to be frightened by menopausal women in films and dramas, many of whom are unrealistic and exaggerated… I also told them that in addition to many physical symptoms, women in the menopausal transition period become psychologically more vulnerable and sensitive. Therefore, I hope they can be more tolerant and caring. At the same time, I myself will try my best to complete this stage of transition smoothly." (Case 10)*

Furthermore, several participants had originally not understood what menopause meant or how it would affect their lives. They were flustered and scared by menopause due to its discrimination and rejection by others. However, through coherent explanations by family members with correct knowledge, such as doctors and daughters, they gained a new perspective on menopause. These participants therefore wielded the correction of inappropriate perceptions as a powerful weapon to combat the stigma of menopause. Accordingly, when some family members used menopause to stigmatize these participants, the latter tried to correctly explain menopause to the former to correct, as much as possible, the misconception that menopause is a mental illness.

#### Subtheme 4: developing a menopause transition management plan

In the fourth subtheme, some participants described the menopausal transition management plans they had developed; their primary reason for developing these plans was as follows: "I want to complete the menopausal transition quickly so that they will stop stigmatizing me." Consequently, the development of a menopausal transition management plan can be considered a strategy for addressing the stigma of menopause.

Some participants also summarized the advice they had received from doctors, family members, friends or colleagues, which they used to develop plans such as travelling, receiving massages, shopping, and using Western medicine and Chinese medicine treatments to treat their menopausal syndrome:

*"I heard from a colleague of mine that she had suffered from menopausal syndrome two years ago and was not feeling well. Then, she went out on a trip for a few days and came back feeling much better. Therefore, I think it is a good idea to travel for a few days, but not until my school is out for the summer and my son is out for the year." (Case 7)*

*"Because I have much discomfort in my cervical spine and joints, my daughter has purchased a massage card so that I can go for a massage to ease my body and relax and unwind. She knows I like new clothes, so to keep me in a happier mood, she gives me a sum of money every month to spend specifically on clothes." (Case 5)*

In addition, once several participants learned that Chinese herbal medicine could help relieve their menopausal syndrome and accelerate their completion of the menopausal transition, they tested it. As one of the participants recounted, *"My daughter came home from work and told me that her colleague's mother also used to have very strong symptoms of menopausal syndrome and was later cured by a doctor in a Chinese hospital. Based on her experience, I also tried Chinese medicine." (Case 11)*

### Theme 4: despair

The last theme describes the sense of despair felt by some of the participants, who tried to cope with the stigma of menopause in their families but did not obtain their desired results. This theme comprises three subthemes.

#### Subtheme 1: deep-rooted perceptions

Some participants had family members who insisted that menopause meant abnormality or could lead to mental illness and felt that they were the only ones who were right, even after the participants had repeatedly explained to them that menopause was just a normal biological phenomenon. This left some participants feeling powerless and hopeless:

*"My husband thinks that my neck pain, leg pain, panic attacks and fatigue are signs of mental illness. I corrected him several times, saying that the doctor and my daughter had said that I was actually going through the menopausal transition, which, like puberty, is something every woman goes through. He looked as if he had heard a joke and did not believe it at all. I did not know what to say to him afterwards; it did not make sense to him at all." (Case 12)*

One participant's husband strongly advocated the idea that menopause can make people mentally ill because he was aware that his colleague's wife had experienced depression and then committed suicide during her menopausal transition: *"He thought his colleague's wife jumped off the building because of menopause… because of her mental problems; so, he thinks that one day I will become like his colleague's wife… It is too depressing and uncomfortable to live with such people every day." (Case 14)*

#### Subtheme 2: restrictions on travel and consumption

Two participants had planned to relieve their menopausal syndrome through massage, shopping and square dancing, but they were strongly impeded by their husbands and were restricted in terms of their freedom to travel and spend money:

*"To relieve my menopausal syndrome and get through the menopausal transition as soon as possible, I planned to go out for regular body massages. However, I had only been there twice when I was stopped by my husband. He felt that massages are only for the rich and I am not entitled to them and told me to go to the massage parlour and withdraw the money from my card..." (Case 5)*

*"Whenever I want to go downstairs to dance, my husband will not let me go out on the grounds that I need to look after my grandchildren. If I say, 'Are not you still at home? 'He goes crazy and scolds me for being irresponsible and says I've become mentally ill because of menopause. In his mind, it does not matter if I die of pain, but my grandson cannot lose a single hair." (Case 9)*

In summary, these participants' husbands objected to their travel and spending mainly on the grounds of saving money and caring for grandchildren. However, it is also clear that they ignored the needs and inner feelings of their menopausal wives, revealing the stigma they attached to menopause. This was so frustrating for the participants that they unanimously stated, *"It is suffocating to live like I’m serving a prison sentence."*

#### Subtheme 3: unknown "healing" times

The final subtheme describes the experiences of some participants who felt hopeless because of their uncertainty about the duration of their menopausal transition. Some relevant excerpts from the interview transcripts are as follows:

*"I asked the doctor when I would recover. However, the doctor was not sure. He just kept prescribing me medication… The medication has side effects, and I've been taking it for over a year with no effect. I doubt that I will ever be cured. Sometimes, I think it would be better to die so I do not have to suffer this." (Case 6)*

*"Going from Western medicine to herbal medicine, I sometimes feel as if I am getting better; however, after a while there is discomfort elsewhere in my body, and it goes back and forth like this, with no hope of getting better." (Case 11)*

*"It is such a desperate feeling—not knowing when it will get better or if it will get better at all." (Case 12)*

In short, these participants' concerns and questions regarding the duration of their 'healing' represent their desperate desire to return to their previous bodily and mental state, partially reflecting the great physical and emotional suffering they have endured within their families. However, as some of the symptoms of menopause can last for a long time and the syndrome’s exact duration individually varies, there is no definitive timeframe. Hence, the participants felt very desperate about their condition.

## Discussion

The aim of this study is to investigate the stigmatization experiences of Chinese menopausal women in their families. We have found that menopausal women suffer great physical and emotional pain and hardship from their families, e.g., their violent treatment and lack of companionship with and attention from family members. They typically attempt to improve this situation, e.g., by trying to correct inaccurate perceptions of menopause among family members. Unfortunately, some of the efforts of the menopausal women in our study failed to produce effective results, leaving them rather hopeless about their current lives.

All of the participants in this study have experienced verbal abuse from their family members for being "mentally ill" or "crazy" or, more seriously, have been beaten by their husbands due to their menopause. The medical definition of menopause, a negative experience entailing numerous and adverse physical and emotional symptoms, may have influenced how menopause is perceived and understood by the general public [44]. Furthermore, in Chinese popular culture, menopause has traditionally been viewed as an entirely negative concept with gendered cultural connotations, often used as a derogatory term to blame or abuse someone or some group of people. Additionally, in popular Chinese literature or films, female menopause is often exaggerated, functioning as an important element in tragic outcomes for individuals and families [36]. The menopausal transition is thus demonized as the most terrifying phase in a woman's life and even considered a time of disaster for family life. Hence, our participants' family members expressed very negative views of menopause, and there was even a degree of self-stigma among most of the participants themselves. This finding therefore confirms that the mass media contributes to the public's understanding of menopause as a negative experience through the dissemination of misinformation [45, 46]. Accordingly, health care providers, in addition to focusing on the physical condition of menopausal women, should disseminate scientific information about menopause to menopausal women and their family members to destigmatize it and reduce the violence related to it as much as possible.

Although most participants experienced a certain degree of self-stigma regarding menopause, some demonstrated relatively scientific knowledge of menopause. This finding may be related to the educational level of the participants. In this study, the education level of most participants was relatively low, and they had received little menopause-related education. A study examining the impact of education on menopausal symptoms and quality of life in Chinese women revealed that those with higher education levels had a better quality of life [47]. Indeed, one theory holds that education is critical for health throughout life because it can persistently affect a series of social, economic and behavioural factors that ultimately influence disease and death [48]. For example, education provides knowledge about the physiological and psychological aspects of menopause, which can guide and change people's attitudes and lifestyles in the face of menopause and improve the quality of life of menopausal women. A study evaluating the effectiveness of health education on menopausal symptoms, knowledge, and attitudes suggested that health education is an effective way to positively change the severity of menopausal symptoms and improve the knowledge and attitudes regarding menopause [49]. It is undeniable that adequate knowledge and a positive attitude towards menopause are critical for women to cope with the changes associated with menopause [50]. Therefore, it is extremely useful and urgent to strengthen public health education regarding menopause.

Notably, although all participants agreed that being treated violently (especially physically) was particularly harmful, some of them expressed a high level of acceptance and tolerance. In particular, some participants suggested that family members, especially their husbands, beat them for their "own good" and "for the sake of the grandchildren." Although they expressed anger and sadness, they also stated that such a violent situation was more acceptable and understandable because it reflected a concern for the greater good. Some previous studies have documented similar findings [51, 52]. Intimate partner violence (IPV) remains widespread in China. A review of IPV in China shows that the lifetime prevalence of psychological, physical, and sexual violence in the general population ranges from 17.4 to 24.5%, 2.5 to 5.5%, and 0.3 to 1.7%, respectively [53]. This effect may be strongly linked to patriarchal male values [54]. To effectively maintain this gender order in patriarchal families, Confucian norms provide greater power to men and emphasize the subordinate position of women, driving unequal gender relations between spouses [55]. This may explain why these participants were treated violently by their husbands but rationalized these acts of violence. In other words, a key reason for the prevalence of intimate partner violence among women is that intimate partner violence is given legitimacy [56]. However, in recent years, with the rapid promotion of women's equal rights and the continuous development of the economy, some traditional Chinese values have been challenged. This change has prompted us to pay more attention to women subjected to violence and deeply reflect on the current values. Thus, it is necessary to provide appropriate resources for counselling and referrals for study participants who have experienced IPV to improve their living environment and promote their physical and mental health development. The All-China Women's Federation, as an important organization that safeguards women's rights and interests and promotes gender equality and women's comprehensive development, should actively provide assistance and support to victims, especially psychological counselling, legal counselling and referral services. Psychological treatment and legal education for the perpetrators of domestic violence should also be provided, and the public security department should be contacted for handling when necessary.

Furthermore, our participants lacked attention and companionship within their families. This mainly manifested in the physical and psychological pain and depression caused by menopause, which was not understood by family members, in the neglect and dislike of performing household chores amid some physical symptoms, and in the difficulty finding family members with whom to discuss their menopausal feelings. Similar experiences have been revealed in other studies. When women’s menopausal transition begins, their husbands usually have little knowledge of the meaning, symptoms, etc., of menopause. Thus, in our study, when these women began to complain of their emotional and physical symptoms brought on by menopause, their husbands did not consider these symptoms real but rather excuses for their wives to complain about their life [57]. In a survey of women's experiences of menopause in Turkey, one respondent stated the following after a conflict with a family member: "My family is uninformed and inconsiderate; they say everyone goes through it, not just you [58].” Several studies have proven that a lack of knowledge about menopause may be an important reason men are unable to understand their wives' postmenopausal symptoms or provide support to them [44, 59]. In China, most men know little about menopause [60], and there are barriers to their access to menopause-related knowledge, e.g., its social stigma, their limited access to information and their difficulty discussing menstruation, consistent with research in the USA and Bangladesh [61, 62]. However, as the most intimate and important person in a woman’s life, spouses should help their wives through the transition period by fully understanding menopause and becoming the most important source of social support. One study claims that training menopausal women's spouses in menopausal symptoms, complications and management can significantly improve women's sense of social support during the menopausal transition [63]. Therefore, efforts to promote menopausal knowledge and humanistic care and improve access to relevant information should be vigorously promoted, with menopause-related education and support services (especially the support of spouses of menopausal women) in the community as one of the key initiatives.

In addition, this study has notably revealed that a lack of attention and companionship from family members is divided into an active or passive lack among family members. An active lack refers to the subjective reluctance of family members to listen or pay attention to participants and the perception that exploring and understanding women's feelings about menopause is unnecessary. This may be due to misconceptions about menopause or the belief that menopause is a private matter for women. In this study, a lack of passivity was mainly due to objective reasons, e.g., their family members being busy with work and not having enough free time to communicate with participants. As China is the world's most populous country, the pressure of competition is enormous, and young people invest much time in their work [64] to obtain a pay raise or promotion, leaving them insufficient space to focus on their families and spend time with them, whether parents or children. Some studies have discussed the topic of work-life imbalance [65–67]; long working hours have been suggested to negatively impact employees' lives, and intertwined work and life has been found to limit the ability to act as effective marriage partners and parents [68]. Vague work and life boundaries seem to have become a new norm. This underscores the need to balance work and family and to pay attention to the needs of menopausal women, who long for understanding and confidence from their family members.

To cope with menopausal stigma, participants adopted four main strategies: keeping quiet, fighting back, changing inappropriate perceptions, and developing a menopausal transition management plan. These are similar to some of the coping strategies for experiencing stigma summarized in a qualitative systematic review [69]. That the participants took steps to cope with menopausal stigma confirms the idea that menopausal women have agency when facing complex life stages [70] and partially reflects the complex dynamics of experiencing menopausal stigma while seeking to complete the process. Critically, a response of silence is a negative one, implying an act of avoidance, surrendering one’s voice in acquiescence to the violence of family members. Previous research has shown that avoidance helps protect a person from the unbearable negative effects that are often caused by shame [71]. This may be why menopausal women adopt this coping strategy. According to Miller, a stigma can be a source of stress for stigmatized individuals, and different individuals experience different emotional, cognitive and behavioural responses [72]. Therefore, it is important to develop effective coping strategies to reduce the consequences of any menopausal stigma for menopausal women.

Despite some efforts to address the stigma of menopause, some participants felt desperate about their situation. They found it difficult to change anyone's perceptions, and even when they explained the science of menopause to their family members repeatedly, some of the latter continued to label the participants “abnormal” or “mentally ill.” In addition, some participants had developed menopausal transition management plans that were resisted by family members on the grounds that they were a waste of money or that they needed to stay home to care for their grandchildren. Finally, several participants highlighted their uncertainty about the duration of their menopausal transition and expressed their lack of hope that they would ever be “cured.” It has been suggested that women with negative experiences or high levels of stress may experience a more negative menopausal transition [73], which we have confirmed in this study. Previous research has also shown that factors such as knowledge and family understanding are critical in helping women manage menopause [74]. Hence, there is a critical need to focus on menopausal women's experiences of stigma and to implement effective anti-stigma interventions by disseminating knowledge about menopause and strengthening family support.

Several limitations of our work should be noted. First, all 14 participants were from Nanjing, a city in eastern China, impeding the generalization of our findings to menopausal women from other regions, who may have different experiences. Second, public interview settings such as Chinese restaurants and parks may have limited participants' responses. This is because, compared to those interviewed in private environments, participants interviewed in public settings may have been less candid about sensitive topics due to concerns about their comments being overheard by others. Finally, the presence of one participant's daughter during her interview may have affected the openness and veracity of this participant's responses. Although the presence of family members can help participants understand the interview better, it may also affect the participant's level of honesty in their narration. For example, cultural taboos may exist for sensitive topics such as physical violence and intimate relationships; thus, the abovementioned participant may not have wanted her daughter to know that she is often subjected to violence by her husband. This may imply that the information provided by the participant was limited, which could have negatively impacted the quality of the data collected. Nonetheless, to minimize these limitations, we selected private and quiet settings for the interviews as much as possible, with participants' agreement. Additionally, after the interview, we requested that the participant who had her daughter present during the interview conduct a separate in-depth interview with us for approximately 30 min to discuss certain topics. These factors should be taken into account when interpreting the findings of this study.

## Conclusions

This study not only provides a context for better understanding the life experiences of Chinese menopausal women in the family but also supplements the relevant research on the stigmatization experiences of Chinese menopausal women in the family and their feelings about these experiences on the basis of current research on physical symptoms, mental health challenges, and available treatment programs among this population. In this study, we adopt a phenomenological approach to describe the stigmatization experiences of menopausal women in the family. We have found that menopausal women are often subjected to verbal and physical violence by family members, such as being insulted as "mentally ill". In addition, menopausal women lack the companionship and attention of their family members. Specifically, they are harmed by not being understood by family members in terms of their physical and psychological pain and depression caused by menopause, neglecting the value of labour, and struggling to find family members to discuss their menopausal feelings with. We have also found that while some menopausal women resort to silent coping strategies when they are verbally abused or beaten by family members, others fight back with verbal warnings or by leaving home. In addition, correcting family members' inappropriate perceptions of menopause and developing a menopausal transition management plan are also strategies for coping with the stigma of menopause. Unfortunately, due to their difficulty changing their family members' perceptions and the restrictions on menopausal women's freedom to travel and consume, as well as the unknown duration of their menopausal transition, some menopausal women feel very hopeless about their lives, and some even become suicidal.

In conclusion, we have demonstrated that the stigmatization of menopause is both a symptom of a broad societal lack of knowledge regarding menopause and a reflection of the patriarchal oppression of women in a particular cultural context. Thus, effective anti-stigma interventions should be implemented to disseminate knowledge about menopause and strengthen family support. The findings of this study will therefore help menopausal women and society in general better understand the stigmatization experiences of menopausal women and amplify their inner voices. They can also serve as a reference for the formulation of menopause-related health policies and the promotion of humanistic care for menopausal women.

## Data Availability

The datasets analysed during this study are available from the corresponding author on reasonable request.
